# Retained wooden splinter in the gluteal region presenting 10 years after initial injury

**DOI:** 10.1093/jscr/rjaf1061

**Published:** 2026-01-22

**Authors:** Ezekiel Aaron, Alec Winder

**Affiliations:** General Surgical PHO, Townsville University Hospital, 100 Angus Smith Drive, Douglas QLD 4814, Australia; General Surgeon, Townsville University Hospital, 100 Angus Smith Drive, Douglas QLD 4814, Australia

**Keywords:** retained wooden foreign body, sinus tract, lumbosacral, gluteal

## Abstract

Delayed presentation of retained foreign bodies with sequelae of chronic inflammation is a rare but well documented phenomenon. Here we present the unusual case of a 23-year-old female with a retained wooden splinter in the buttock region presenting more than 10 years post injury. A foreign body was not identified on initial imaging. Only after surgical exploration and targeted ultrasound was a wooden splinter identified and removed.

## Introduction

Over one-third of penetrating foreign bodies (FB) are not identified on initial assessment [1]. Organic matter like wood and vegetation are radiolucent so can be missed on plain radiography [1]. However, they are highly reactive to surrounding tissue and can cause a local inflammatory response. Consequently, patients can present many years after their initial trauma with chronic pain, swelling, and recurrent infections [2–4].

An inflammatory granuloma can form which appears as a pseudotumor in soft tissue on cross-sectional imaging [5]. Ultrasound (US) is the most cost-effective, accessible and sensitive imaging modality available for detecting organic foreign bodies. On US, wood appears hyperechoic with surrounding hypoechoic areas of soft tissue inflammation [4, 6].

In the following case, despite initial negative imaging, a FB was found after US guided surgical exploration.

## Case report

A 23-year-old female was initially referred to the neurosurgical clinic with long-standing radicular lower back pain. The patient believed it was a chronic musculoskeletal injury from falling on a wooden stump 10 years ago. The pain had been increasing in severity over the last few years, exacerbated by backpacking activities.

**Figure 1:**
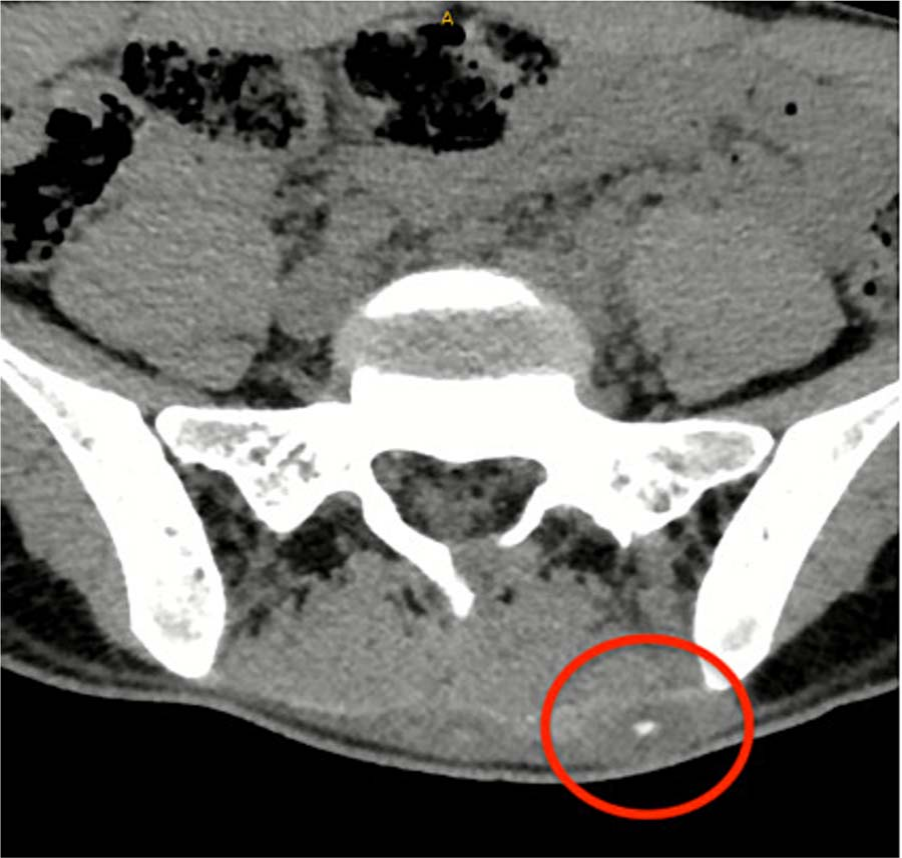
CT lumbosacral spine showed a left paraspinal calcified haematoma.

A CT lumbosacral spine was performed which demonstrated no spinal pathology but detected a left paraspinal calcified hematoma which was thought to be the cause of her symptoms and so was referred to the general surgeons (Fig. 1).

**Figure 2:**
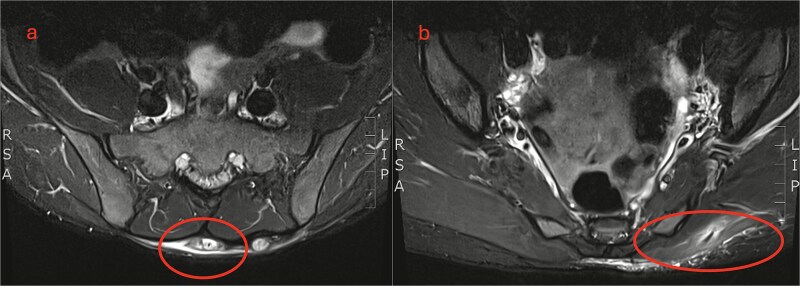
MRI lumbosacral spine. (a) Axial view shows a soft tissue mass overlying the sacrum. (b) Sagittal view shows a linear extension tracking down to the left gluteus maximus.

In the general surgery clinic, a 2–3 cm sized swelling over the lumbar region was palpated. Magnetic resonance imaging (MRI) showed a chronic subcutaneous soft tissue mass overlying the sacrum. They also noted a linear extension of similar signal characteristics extending to the left gluteus maximus muscle (Fig. 2).

Ultimately, surgery was performed to excise this lumbosacral lesion. Intraoperatively, scar tissue was found with surrounding purulent fluid. This was associated with a sinus tract extending deep into the left gluteus maximus on probing, raising the suspicion of an occult foreign body. Additional imaging was obtained before further wound exploration. Histology confirmed microscopic fragments of foreign material with surrounding scar tissue and inflammatory exudate.

After the operation, with further careful history taking, the patient recalled some of the wooden splinters had penetrated her buttock region. She believed she had removed all of them, hence she did not present for a medical review at the time.

**Figure 3:**
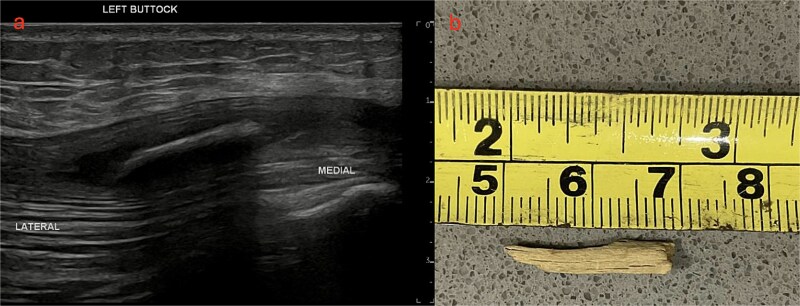
US left buttock and photo of wooden splinter (a) US left buttock shows a 20 × 2 mm hyperdense foreign body within the left gluteus maximus 12 mm deep. (b) Photo of wooden splinter after retrieval.

A targeted US was performed, showing a 20 × 2 mm FB buried within the left gluteus maximus, 12 mm deep. On surgical re-exploration, the gluteus muscle was opened over the US guided markings. A sinus tract was identified which communicated with the previously identified lumbosacral tract. A strip of gauze was threaded through the tract and flossed to remove a fragment of wood (Fig. 3).

She was discharged the following day after the removal of her drain. She was seen in the outpatient clinic 3 weeks later. At this point her sutures had been removed, and her wound had healed completely. She had no more pain and was discharged from general surgical follow up.

## Discussion

Studies have reported 38% of foreign bodies are missed on initial examination [1]. Patients can present years after their injury with chronic pain, a lump or recurrent abscesses [5]. In this case the patient had ongoing radicular lower back pain leading her to see a specialist 10 years later.

Wood is highly reactive and inflammatory compared to other inorganic material and forms a localized inflammatory reaction with surrounding tissue [3, 4]. They can form lytic lesions, periosteal reactions, pseudotumours in soft tissue and sinus tracts [5, 7–9]. In this case, chronic inflammation from a retained wooden splinter had formed a sinus tract in the left gluteus maximus extending up to the lumbosacral region (Fig. 3). On surgical exploration, purulent material and thickened, fibrous tissue was found surrounding the tract indicating a chronic infectious / inflammatory process.

The best imaging modalities for organic material is US /MRI [4, 6, 10]. However, MRI is costly, and not readily available so is not recommended for initial assessment [4, 10]. US is easier to access and portable but can be operator dependent [4]. Wood is usually hyperechoic with a surrounding hypoechoic halo from the acoustic shadowing and the inflammatory response [4] (Fig. 3). In this case, the patient had a CT and MRI which demonstrated a soft tissue mass in the lumbosacral region but no foreign body (Figs 1 and 2). With low clinical suspicion, radiologists are less likely to detect a FB or the secondary features of one. In retrospect, the soft tissue mass seen on MRI in the lumbosacral region extending down to the left gluteus maximus corresponds to the sinus tract found intraoperatively. The central T2 hypointense / signal void region represents the wooden splinter which attenuated as it absorbed fluid from the surrounding tissue (Fig. 2).

## Conclusion

This case highlighted the importance of maintaining a high clinical suspicion for retained FB in patients presenting with localized symptoms with a history of trauma especially with wood or other organic material. Retained foreign bodies should be kept a differential despite negative cross-sectional imaging and time since injury. An US should be performed during the diagnostic process.

## References

[1] Anderson MA, Newmeyer WL, Kilgore ES Jr. Diagnosis and treatment of retained foreign bodies in the hand. Am J Surg 1982;144:63–7. 10.1016/0002-9610(82)90603-1 7091533

[2] Imre E . Extremity foreign body injury presenting years after trauma: 2 case reports and review of literature. EJMCR. 2021;5:337–40. 10.24911/ejmcr/173-1624387725

[3] Choi JH, Oh SS, Hwang JH, et al. Residual foreign body inflammation caused by a lumbar beam penetrating the facial region: a case report. ACFS 2023;24:37–40. 10.7181/acfs.2022.01018 36858360 PMC10009212

[4] Graham DD Jr . Ultrasound in the emergency department: detection of wooden foreign bodies in the soft tissues. J Emery Med 2002;22:75–9. 10.1016/s0736-4679(01)00440-1 11809560

[5] Andrea C . Wooden foreign bodies in the foot. AJS 1980;140:585–7. 10.1016/0002-9610(80)90218-4 (9 October 2025, date last accessed). 6999928

[6] Rupert J, Honeycutt JD, Odom MR. Foreign bodies in the skin: evaluation and management. Am Fam Physician 2020;101:740–7. 32538598

[7] Kartiko M, Guduru M, Denotter T, et al. Occult traumatic impaled foreign body, a challenging diagnosis of severe chronic lower extremity radicular pain. Trauma Case Rep 2021;35:100514. 10.1016/j.tcr.2021.100514 34409146 PMC8361320

[8] Gandham E-J, Tyagi A, Prabhu K. An unusual cause of cervical radicular pain-foreign body in Esophagus. Iran J Otorhinolaryngol 2018;30:237–9. https://pmc.ncbi.nlm.nih.gov/articles/PMC6064759/ (10 October 2025, date last accessed). 30083531 PMC6064759

[9] Floman Y, Katz S. Osseous lesion simulating a bone tumour due to an unsuspected fragment of wood in the foot. Injury 1975;6:344–5. 10.1016/0020-1383(75)90189-8 1095477

[10] Peterson JJ, Bancroft LW, Kransdorf MJ. Wooden foreign bodies: imaging appearance. AJR 2012;178:557–62. 10.2214/ajr.178.3.1780557 11856673

